# Supplementary material for: Inadequate Vitamin C Intake and Intestinal Inflammation Are Associated with Multiple Micronutrient Deficiency in Young Children: Results from a Multi-Country Birth Cohort Study
Source: Nutrients. 2022 Mar 28;14(7):1408. doi: 10.3390/nu14071408 (PMC9003322; doi:10.3390/nu14071408)
Supplement: Supplementary file 1 [file nutrients-14-01408-s001.zip › nutrients-1611003-supplementary.pdf]

Supplementary file

Table S1. Descriptive characteristics of the study participants, overall and by MAL-ED study sites.

	BGD	BRF	INV	NPB	PEL	PKN	TZH	Overall
	(n=174)	(n=128)	(n=223)	(n=115)	(n=150)	(n=151)	(n=152)	(n=1093)
**Incidence of diarrhea, Mean ± SD**	13.6 ± 10.3	2.29 ± 2.85	6.73 ± 7.04	11.2 ± 10.7	18.4 ± 14.7	31.9 ± 30.1	3.17 ± 2.97	12.3 ± 16.8
**Incidence of cough, Mean ± SD**	92.6 ± 46.7	10.5 ± 9.35	36.2 ± 24.6	30.6 ± 29.0	81.4 ± 41.3	87.0 ± 56.5	10.7 ± 7.17	51.2 ± 48.3
**Incidence of fever, Mean ± SD**	22.7 ± 10.2	1.70 ± 1.63	20.4 ± 9.77	17.5 ± 9.82	15.3 ± 7.54	43.9 ± 26.5	7.46 ± 5.22	19.0 ± 17.3
**Maternal BMI, Mean ± SD**	22.4 ± 3.45	26.2 ± 4.44	22.0 ± 3.98	25.0 ± 3.26	24.9 ± 3.64	21.7 ± 3.68	22.9 ± 2.95	23.4 ± 3.98
**Hands wash after helping child defecate, n (%)**								
**Never**	11 (6.32%)	0 (0%)	77 (34.5%)	1 (0.870%)	0 (0%)	0 (0%)	6 (3.95%)	95 (8.69%)
**Rarely**	15 (8.62%)	2 (1.56%)	42 (18.8%)	2 (1.74%)	2 (1.33%)	4 (2.65%)	41 (27.0%)	108 (9.88%)
**Sometimes**	20 (11.5%)	52 (40.6%)	19 (8.52%)	13 (11.3%)	21 (14.0%)	58 (38.4%)	83 (54.6%)	266 (24.3%)
**Always**	128 (73.6%)	74 (57.8%)	85 (38.1%)	99 (86.1%)	127 (84.7%)	89 (58.9%)	22 (14.5%)	624 (57.1%)
**Hands wash before preparing food, n (%)**								
**Never**	81 (46.6%)	0 (0%)	148 (66.4%)	9 (7.83%)	2 (1.33%)	0 (0%)	6 (3.95%)	246 (22.5%)
**Rarely**	24 (13.8%)	2 (1.56%)	47 (21.1%)	13 (11.3%)	1 (0.667%)	9 (5.96%)	34 (22.4%)	130 (11.9%)
**Sometimes**	38 (21.8%)	72 (56.3%)	7 (3.14%)	34 (29.6%)	36 (24.0%)	74 (49.0%)	82 (53.9%)	343 (31.4%)
**Always**	31 (17.8%)	54 (42.2%)	21 (9.42%)	59 (51.3%)	111 (74.0%)	68 (45.0%)	30 (19.7%)	374 (34.2%)
**Hands wash after using the toilet, n (%)**								
**Never**	8 (4.60%)	0 (0%)	70 (31.4%)	0 (0%)	0 (0%)	0 (0%)	4 (2.63%)	82 (7.50%)
**Rarely**	12 (6.90%)	0 (0%)	15 (6.73%)	1 (0.870%)	3 (2.00%)	3 (1.99%)	31 (20.4%)	65 (5.95%)
**Sometimes**	20 (11.5%)	78 (60.9%)	15 (6.73%)	3 (2.61%)	14 (9.33%)	55 (36.4%)	69 (45.4%)	254 (23.2%)
**Always**	134 (77.0%)	50 (39.1%)	123 (55.2%)	111 (96.5%)	133 (88.7%)	93 (61.6%)	48 (31.6%)	692 (63.3%)
**Use toilet paper, n (%)**								
**Never**	112 (64.4%)	2 (1.56%)	221 (99.1%)	106 (92.2%)	3 (2.00%)	151 (100%)	24 (15.8%)	619 (56.6%)
**Rarely**	11 (6.32%)	2 (1.56%)	2 (0.897%)	3 (2.61%)	2 (1.33%)	0 (0%)	40 (26.3%)	60 (5.49%)
**Sometimes**	24 (13.8%)	19 (14.8%)	0 (0%)	5 (4.35%)	28 (18.7%)	0 (0%)	39 (25.7%)	115 (10.5%)

**Always**	27 (15.5%)	105 (82.0%)	0 (0%)	1 (0.870%)	117 (78.0%)	0 (0%)	49 (32.2%)	299 (27.4%)
**Separate space for kitchen, n (%)**	13 (7.51%)	113 (88.3%)	133 (59.6%)	77 (67.0%)	114 (76.0%)	41 (27.2%)	61 (40.1%)	552 (50.5%)
**Asset score, Mean ± SD**	3.36 ± 1.75	6.82 ± 1.08	4.19 ± 2.05	5.83 ± 1.56	4.95 ± 1.47	2.90 ± 2.22	2.28 ± 1.65	4.20 ± 2.26
**Improved water, n (%)**	174 (100%)	128 (100%)	223 (100%)	115 (100%)	134 (89.3%)	151 (100%)	81 (53.3%)	1006 (92.0%)
**Maternal education (years), Median [IQR]**	5 [2, 7]	9 [8, 12]	8 [4, 9]	9 [5, 10]	7.50 [6, 10]	2 [0, 5]	7 [3, 7]	7 [4, 9]
**Monthly family income (USD), Median [IQR]**	124 [85.9, 182]	334 [310, 386]	81.0 [55.1, 113]	155 [102, 226]	113 [75.9, 158]	144 [90, 212]	18 [12, 36]	112 [60.5, 191]
**Improved sanitation, n (%)**	174 (100%)	128 (100%)	118 (52.9%)	115 (100%)	52 (34.7%)	111 (73.5%)	14 (9.21%)	712 (65.1%)
**Household animal exposure, n (%)**	6 (3.45%)	1 (0.781%)	14 (6.28%)	37 (32.2%)	64 (42.7%)	102 (67.5%)	142 (93.4%)	366 (33.5%)
**Household crowding index, n (%)**								
**Low (0 - 1 people)**	4 (2.30%)	9 (7.03%)	2 (0.897%)	10 (8.70%)	6 (4.00%)	9 (5.96%)	12 (7.89%)	52 (4.76%)
**Medium (2 - 4 people)**	132 (75.9%)	107 (83.6%)	113 (50.7%)	102 (88.7%)	102 (68.0%)	87 (57.6%)	120 (78.9%)	763 (69.8%)
**High (>4 people)**	38 (21.8%)	12 (9.38%)	108 (48.4%)	3 (2.61%)	42 (28.0%)	55 (36.4%)	20 (13.2%)	278 (25.4%)

Table S2. Numeric results for the associations depicted in Figure 4. Presented are the median effects (95% credibility interval) of each predictor on each response variable adjusted for other associations within the model. Effects are expressed as odds for binary responses; otherwise they are expressed as linear effects.

**Response**	**Predictor variables**	**Posterior quantile**		
		**Median**	**[95% Credibility Interval]**	
		**linear effect**		
**Protein intake**	Monthly family income (USD)	0.056	0.005	0.107
	Maternal education (years)	0.072	0.027	0.117
	Crowding [Ref: Low (0-1 people)]			
	Medium (2 - 4 people)	-0.158	-0.322	0.005
	High (>4 people)	-0.225	-0.411	-0.044
**Iron intake**	Monthly family income (USD)	0.028	-0.013	0.069
	Maternal education (years)	0.034	0.000	0.068
**Zinc intake**	Monthly family income (USD)	0.060	0.020	0.099
	Maternal education (years)	0.070	0.035	0.105
	Crowding [Ref: Low (0-1 people)]			
	Medium (2 - 4 people)	-0.033	-0.159	0.093
	High (>4 people)	-0.080	-0.222	0.062
**Thiamine intake**	Monthly family income (USD)	0.071	0.014	0.128
	Maternal education (years)	0.045	-0.004	0.092
**Niacin intake**	Monthly family income (USD)	0.068	0.025	0.113
	Maternal education (years)	0.069	0.031	0.107
**Riboflavin intake**	Monthly family income (USD)	0.057	-0.002	0.114
	Maternal education (years)	0.057	0.006	0.109
	Crowding [Ref: Low (0-1 people)]			
	Medium (2 - 4 people)	-0.291	-0.476	-0.105
	High (>4 people)	-0.339	-0.551	-0.131

**Vitamin-A intake**	Monthly family income (USD)	0.028	-0.011	0.067
	Maternal education (years)	0.043	0.010	0.077
**Vitamin-C intake**	Crowding [Ref: Low (0-1 people)]			
	Medium (2 - 4 people)	-0.115	-0.305	0.071
	High (>4 people)	-0.222	-0.432	-0.017
**Vitamin-B12 intake**	Monthly family income (USD)	0.013	-0.030	0.055
	Maternal education (years)	0.022	-0.015	0.060
	Crowding [Ref: Low (0-1 people)]			
	Medium (2 - 4 people)	-0.169	-0.303	-0.035
	High (>4 people)	-0.229	-0.383	-0.079
**Bacterial load**	Maternal education (years)	-0.052	-0.112	0.009
	Crowding [Ref: Low (0-1 people)]			
	Medium (2 - 4 people)	0.363	0.127	0.592
	High (>4 people)	0.453	0.195	0.712
	Separate space for kitchen	-0.100	-0.223	0.023
	Improved sanitation	-0.118	-0.275	0.038
	Hands wash after helping child defecate (Ref: Never)			
	Rarely	-0.037	-0.307	0.223
	Sometimes	-0.132	-0.404	0.144
	Always	-0.240	-0.509	0.031
	Hands wash after using the toilet (Ref: Never)			
	Rarely	-0.129	-0.434	0.187
	Sometimes	-0.042	-0.334	0.248
	Always	-0.050	-0.309	0.216
**Viral load**	Monthly family income (USD)	-0.114	-0.194	-0.033
	Separate space for kitchen	-0.130	-0.276	0.018
	Improved sanitation	-0.108	-0.281	0.069
	Hands wash after helping child defecate (Ref: Never)			
	Rarely	0.271	-0.009	0.563
	Sometimes	0.132	-0.157	0.425
	Always	-0.001	-0.266	0.268
**Parasite load**	Monthly family income (USD)	-0.016	-0.091	0.057
	Maternal education (years)	-0.111	-0.177	-0.046
	Crowding [Ref: Low (0-1 people)]			
	Medium (2 - 4 people)	0.025	-0.218	0.271
	High (>4 people)	0.208	-0.067	0.480
	Separate space for kitchen	-0.128	-0.257	0.005
	Improved sanitation	-0.195	-0.439	0.049
	Hands wash after helping child defecate (Ref: Never)			
	Rarely	0.084	-0.189	0.349
	Sometimes	0.112	-0.168	0.391
	Always	-0.029	-0.309	0.252
	Hands wash after using the toilet (Ref: Never)			
	Rarely	-0.062	-0.395	0.262
	Sometimes	-0.105	-0.408	0.192
	Always	-0.183	-0.461	0.091
	Use toilet paper (Ref: Never)			
	Rarely	-0.296	-0.584	-0.004
	Sometimes	-0.157	-0.409	0.087
	Always	-0.242	-0.504	0.010
**Incidence of diarrhea**	Parasite load	0.059	-0.005	0.122
**Incidence of fever**	Parasite load	0.019	-0.039	0.073

	Viral load	-0.054	-0.100	-0.009
**MPO**	Protein intake	-0.473	-0.693	-0.247
	Iron intake	0.068	-0.109	0.238
	Zinc intake	0.256	-0.039	0.555
	Thiamine intake	0.051	-0.052	0.154
	Niacin intake	-0.115	-0.314	0.076
	Riboflavin intake	0.053	-0.069	0.171
	Vitamin-A intake	-0.169	-0.416	0.077
	Vitamin-C intake	-0.020	-0.101	0.062
	Vitamin-B12 intake	0.026	-0.181	0.228
	Bacterial load	0.076	0.015	0.136
	Parasite load	0.010	-0.050	0.070
	Incidence of fever	-0.058	-0.136	0.016
	Incidence of diarrhea	-0.171	-0.235	-0.108
			**Odds ratio**	
**MMD**	Female child	0.72	0.54	0.92
	MPO	1.31	1.08	1.51
	Vitamin-C intake	0.70	0.48	0.94
